# Surveillance for adverse events following use of live attenuated chikungunya vaccine, United States, 2024, and the associated public health response in 2024 and 2025

**DOI:** 10.2807/1560-7917.ES.2025.30.32.2500543

**Published:** 2025-08-14

**Authors:** Susan L Hills, Rebekah A Sutter, Elaine R Miller, Edwin J Asturias, Lin H Chen, Beth P Bell, Michael M McNeil, Jeffrey Rakickas, Melinda Wharton, Sarah Meyer, J Erin Staples

**Affiliations:** 1 Centers for Disease Control and Prevention, Fort Collins, Colorado, United States; 2 Centers for Disease Control and Prevention, Atlanta, Georgia, United States; 3 University of Colorado, Aurora, Colorado, United States; 4 Mount Auburn Hospital, Cambridge, Massachusetts, United States; 5 Harvard Medical School, Boston, Massachusetts, United States; 6 University of Washington, Seattle, Washington, United States

**Keywords:** chikungunya, vaccine, adverse event, serious, ixchiq, immunisation, live attenuated

## Abstract

A live attenuated chikungunya vaccine (IXCHIQ) received licensure in the United States (US) for ≥ 18-year-olds in November 2023. Post-licensure safety surveillance identified 28 adverse events in 2024 among US persons, including six neurological or cardiac serious adverse events (SAEs) in males ≥ 65 years. In early 2025, providers were alerted, a precaution for older persons was issued and vaccine guidance was updated. In May, following 11 additional SAEs reported outside the US, use in older persons was temporarily paused until 6 August 2025.

A live attenuated chikungunya vaccine (CHIK-LA), manufactured by Valneva as IXCHIQ, was licensed in the United States (US) in November 2023 for individuals aged ≥ 18 years [1]. Post-licensure, safety surveillance was conducted through the Vaccine Adverse Event Reporting System (VAERS), a national spontaneous reporting system, co-managed by the Centers for Disease Control and Prevention (CDC) and US Food and Drug Administration (FDA). It is designed to detect rare or previously unreported adverse events (AEs) or changes in reporting patterns that might signal a safety concern [2]. The FDA regulatory definitions are used to classify AEs as non-serious or serious [3]. We reviewed VAERS reports and conducted enhanced surveillance to obtain additional clinical and outcome data from patient and provider interviews. We discussed serious AEs (SAEs) with vaccine safety experts from CDC's Clinical Immunization Safety Assessment (CISA) project, and calculated SAE rates [4]. The CDC reviewed this activity and deemed it public health surveillance based on the definition in the US Code of Federal Regulations [5]. Our aim was to better understand the AEs after administration of this newly licensed vaccine to inform any needed public health action. 

## Summary of adverse events reports from post-marketing safety surveillance

Overall, 28 AEs were reported following CHIK-LA administered during 2024 in the United States, including 22 reports for non-serious and six reports for serious events. Two additional reported events were excluded because one was from a location outside the US, and one was incorrectly reported (Guillain–Barré syndrome in an individual who had not received CHIK-LA). The first AE report to VAERS on 6 May 2024 was for a non-serious AE; the non-serious AEs are summarised in Table 1, while the SAEs are summarised with more details in Table 2 and Table 3.

**Table 1:** Non-serious adverse events after administration of live attenuated chikungunya vaccine, United States, 2024 (n = 22)

Event type	n
Arthralgia with other systemic symptoms (e.g. fever, myalgia, headache, rash, fatigue)	11
Fever with headache or malaise	4
Generalised myalgia	2
Dizziness and nausea	1
Headache, arm pain and paraesthesia	1
Flushing	1
Sore throat	1
Syncope	1

Events reported to the Vaccine Adverse Event Reporting System (VAERS); the first event occurred in May 2024. Each of the 22 individuals experienced one of these event types.

**Table 2:** Demographics, medical history, vaccination details and outcomes for patients experiencing serious adverse events following administration of live attenuated chikungunya vaccine, United States, 2024 (n = 6)

**Patient**	**Age group (years)**	**Main medical comorbidities**	**Co-administered vaccines**	**Symptom onset after vaccination (days)**	**Duration of hospitalisation**	**Discharge diagnosis(es)**	**Period to full recovery**
1	75–84	Coronary artery disease Chronic heart failure Chronic kidney disease Hypertension Hyperlipidaemia Chronic thrombocytopenia	None	3	2 days	Encephalopathy and generalised weakness	3.5 weeks
2	75–84	IgA deficiency Coronary artery disease Hypertension Hyperlipidaemia Hypothyroidism Benign prostatic hyperplasia	Japanese encephalitis vaccine (inactivated Vero cell culture-derived)	4	7 days Readmitted 4 days post discharge with urinary retention and discharged after 1 day	Acute metabolic encephalopathy with hyponatraemia, hypokalaemia, and hypochloraemia and resolved fever of unknown origin	Patient reported improving symptoms but some ongoing weakness and balance issues at 7 months post discharge
3	85–94	Diabetes mellitus type 2 Heart failure Hypertension Hypothyroidism Anaemia Hyperlipidaemia	None	3	23 days	Toxic metabolic encephalopathy and fever possibly related to post-vaccination inflammatory response with possible superadded bacterial pneumonia	Patient reported some persistent weakness but mostly recovered at 1 month post discharge
4	65–74	Prostate cancer Hypothyroidism Hypertension Dyslipidaemia	None^a^	5	3 days	Aseptic meningitis	1 month
5	65–74	Hyperlipidaemia	Typhoid vaccine (live oral)^b^	4	2 days	Atrial flutter with rapid ventricular response and suspected small non-ST-segment elevation myocardial infarction	2 days
6	65–74	Ischaemic cardiomyopathy Coronary artery disease Hypotension Chronic leukopenia Chronic thrombocytopenia	Japanese encephalitis vaccine (inactivated Vero cell culture-derived)	3	Not hospitalised^c^	Worsened and prolonged hypotension on the background of pre-existing cardiomyopathy and hypotension	2 weeks

^a^ Received six inactivated vaccines at intervals 8–15 day earlier including Tdap (Tetanus, diphtheria, pertussis), influenza, polio, typhoid, Vero cell-derived Japanese encephalitis, and hepatitis A vaccines.

^b^ Received COVID-19 vaccine and high dose influenza vaccine 19 days prior.

^c^ Classified as ‘other important medical event’ which is a serious adverse event under United States Food and Drug Administration regulatory definition [3].

Events reported to the Vaccine Adverse Event Reporting System (VAERS); all chikungunya vaccine doses were administered to patients during pre-travel consultations and the first event occurred in June 2024. All six patients were male.

**Table 3:** Key laboratory, infectious disease testing and imaging results for patients experiencing serious adverse events following administration of live attenuated chikungunya vaccine, United States, 2024 (n = 6)

Patient	General laboratory findings	Infectious disease testing	Imaging
1	Leukopenia: 2,100 white blood cells/µL Acute on chronic thrombocytopenia: 29,000 platelets/µL^a^ Elevated AST: 120 units/L Elevated ALT: 124 U/L Elevated bilirubin: 1.7 mg/dL Hyponatraemia: 132 mmol/L	COVID-19, influenza, RSV PCR test: negative Blood culture: no growth	Brain MRI: No acute changes Head CT: No acute changes
2	Thrombocytopenia: 138,000 platelets/µL Elevated ALT: 93 U/L Hyponatraemia: 129 mmol/L Hypokalaemia: 2.8 mmol/L Hypochloraemia: 95 mmol/L	COVID-19 PCR test: negative Influenza A/B antigen test: negative Blood cultures: no growth	Brain MRI: No acute intracranial abnormalities Chest X-ray: no acute abnormalities
3	Anaemia (chronic): 8.8 g/dL Hyponatraemia: 118 mmol/L Hypochloraemia: 85 mmol/L Hypocalcaemia: 7.6 mg/dL Decreased albumin: 2.3 g/dL Elevated AST: 68 U/L Elevated ALT: 189 U/L Elevated proBNP: 779 pg/mL	Chikungunya virus ribonucleic acid detected in serum by RT-PCR (day 13)^b^	Head CT: No acute abnormalities Chest X-ray: mild right infrahilar infiltrate worsening to bilateral infiltrates Echocardiogram: small pericardial effusion
4	CSF pleocytosis: 109 white cells/µL (35% neutrophils), 157 red cells/µL. Hyponatraemia: 129 mmol/L	Meningoencephalitis CSF PCR panel^c^: negative Respiratory PCR panel: negative Chikungunya IgM and neutralising antibodies detected in CSF, chikungunya virus ribonucleic acid not detected (day 12)^b^ CSF culture: no growth	Brain MRI: no acute abnormalities Brain CT: no acute abnormalities
5	Leukopenia: 3,100 white blood cells/µL Hypocalcaemia: 7.7 mg/dL Elevated troponin: 594 Elevated proBNP: 3,282 pg/mL	COVID-19, influenza and RSV PCRs: negative	Nuclear medicine stress test: suspicious for small infarct involving distal anterior wall Transthoracic echocardiogram: septal hypokinesis CT angiogram: no pulmonary embolus
6	Acute on chronic thrombocytopenia: 72,000 platelets/µL Acute on chronic leukopenia: 2,700 cells/µL Elevated AST: 67 U/L		

ALT: alanine transaminase; AST: aspartate aminotransferase; CSF: cerebrospinal fluid; CT: computed tomography scan; MRI: magnetic resonance imaging; proBNP: N-terminal B-type natriuretic peptide; RSV: respiratory syncytial virus; RT-PCR: reverse transcription PCR.

^a^ A platelet transfusion followed by lumbar puncture was recommended but the patient declined.

^b^ All chikungunya laboratory tests (i.e. RT-PCR, IgM, and neutralising antibody assays) were conducted at the United States Centers for Disease Control and Prevention with laboratory-developed tests.

^c^ Meningoencephalitis CSF panel: PCR for *Haemophilus influenzae* type b, *Listeria monocytogenes*, *Neisseria meningitidis*, *Streptococcus pneumoniae*, cytomegalovirus, enterovirus, herpes simplex virus type 1 and 2, human herpesvirus 6, human parechovirus, varicella zoster virus, cryptococcus, *Escherichia coli.*

Events reported to the Vaccine Adverse Event Reporting System (VAERS) after administration of live attenuated chikungunya vaccine during 2024.

Normal ranges: albumin: 3.4–5.5 g/dL; ALT: 10–40 units/L; AST: 15–37 units/L; bilirubin: 0.1–1 mg/dL; chlorine: 98–110 mmol/L; haemoglobin: 11.8–16.1 g/dL; calcium: 8.5–10.5mg/dL; sodium: 136–145 mmol/L; proBNP: < 125pg/mL for ages < 75 years and < 450 pg/mL for ages ≥ 75 years; platelets: 150,000–450,000/µL; potassium: 3.5–5.3 mmol/L; troponin: ≤ 41.0 ng/L; white blood cells: 4,000–10,000 cells/µL.

### Patient 1

A male in the age group 75–84 years with multiple comorbidities was vaccinated with CHIK-LA and 3 days later developed myalgia, arthralgia, fever, chills, headache, weakness, anorexia, severe fatigue and unsteady gait. Seven days after vaccination he sought medical care and was diagnosed with acute kidney injury, probably dehydration-related. Brain imaging identified no acute changes. On day 11, he was hospitalised with myalgia, fatigue and weakness in the left lower extremity mainly related to hip flexor weakness. Investigations indicated no specific aetiology, and the hospital records noted a discharge diagnosis of encephalopathy and generalised weakness.

### Patient 2

A male in the age group 75–84 years with asymptomatic selective IgA deficiency and other comorbidities was vaccinated with CHIK-LA and Vero cell culture-derived Japanese encephalitis vaccine (JE-VC). Four days later he developed severe fatigue, fever, diarrhoea, myalgia and urinary urgency. On day 6, he sought care, and an unspecified viral illness was diagnosed. On day 8, he presented to a hospital with profound weakness and intermittent confusion and was admitted with fever of unknown origin, diarrhoea, dehydration, hyponatraemia and hypochloraemia. A suspected urinary tract infection was treated with antibiotics, although the patient reported no dysuria or haematuria and a urine analysis was unremarkable. Investigations did not reveal the cause of the symptoms, and hospital records noted a discharge diagnosis of acute metabolic encephalopathy and resolved fever of unknown origin.

### Patient 3

A male in the age group 85–94 years with an extensive medical history was vaccinated with CHIK-LA and 3 days later developed lethargy, drowsiness and fever, followed by a deterioration of mental status and mild dyspnoea. By day 8, he had extreme confusion and was hospitalised with hyponatraemia-related acute encephalopathy and dyspnoea. Investigations demonstrated multiple metabolic abnormalities and mild pulmonary infiltrates which worsened during hospitalisation. Chikungunya virus ribonucleic acid was detected in a serum sample taken on day 13. The reported discharge diagnosis was toxic metabolic encephalopathy and fever.

### Patient 4

A male in the age group 65–74 years with several comorbidities was vaccinated with CHIK-LA. He had received six other inactivated vaccines 8–15 days earlier (Table 2). Initial symptoms commenced 5 days after CHIK-LA vaccination and included fever, headache, fatigue and myalgia, progressing to photophobia and neck stiffness. He was hospitalised on day 12 with aseptic (i.e. non-bacterial) meningitis. Testing identified no infectious aetiologies but anti-chikungunya virus IgM and neutralising antibodies were detected in cerebrospinal fluid.

### Patient 5

A male in the age group 65–74 years with hyperlipidaemia was vaccinated with CHIK-LA and live oral typhoid vaccine. He had received two other vaccines 19 days earlier (Table 2). He developed myalgia and fever 4 days after CHIK-LA vaccination, followed 2 days later by tachycardia and palpitations. On day 8, he was hospitalised. An electrocardiogram indicated atrial flutter with rapid ventricular response, and elevated troponin and a nuclear stress test suggested a small non-ST-segment elevation myocardial infarction.

### Patient 6

A male in the age group 65–74 years with multiple comorbidities, including hypotension probably secondary to underlying cardiac disease, was vaccinated with CHIK-LA and JE-VC. Three days later, he experienced fatigue, weakness, light-headedness and mild dyspnoea. On presentation to his physician 8 days after vaccination, the patient’s systolic blood pressure was 30 points lower than his baseline and had not returned to normal when he presented again 1 week later. The event was described as an episode of worsened and prolonged hypotension.

## Expert consultation and calculation of severe adverse event rates

Consultants from CISA with expertise in vaccine safety, infectious diseases, geriatrics, cardiology and neurology reviewed the SAEs. For each report, at least one expert considered that an association with CHIK-LA was plausible. 

We calculated SAE rates using data from IQVIA, a company that provides healthcare data. The data included those on estimated sales (all settings) which, per IQVIA, capture ca 90% of doses sold, and on doses administered by age group (retail pharmacies only) [6]. Based on estimates of 13,891 doses sold (unadjusted for incomplete capture) during 2024, and 52.7% of CHIK-LA doses administered to persons aged ≥ 65 years, the estimated rates of SAEs and hospitalisations among persons aged ≥ 65 years were, respectively, 82 per 100,000 (95% confidence interval (CI): 30–180/100,000) and 68 per 100,000 (95% CI: 20–160/100,000).

## Discussion

The FDA licensed CHIK-LA in November 2023 following a detailed review of immunogenicity and safety data. In the pivotal phase 3 trial, 3,082 subjects were included in the vaccine arm for the safety assessment, including 287 aged 65–74 years and 59 aged ≥ 75 years, and 1,033 were in the placebo arm [7,8] Two vaccine-related SAEs were reported including hospitalisations for severe myalgia in a 58-year-old female with a history of fibromyalgia, migraines and hypertension, and for atrial fibrillation and hypovolaemic hyponatraemia in a 66-year-old male with hypertension. Following distribution of CHIK-LA in the United States in 2024, six reported neurologic or cardiac SAEs occurred. All six SAEs occurred within 3–5 days of vaccination in males aged ≥ 65 years, most of whom had multiple underlying medical conditions.

Determining causality from VAERS data is generally not possible, in part because of frequent concomitant or recent administration of other vaccines or medications, variable report quality, occasional inadequacy of clinical investigations, and lack of an unvaccinated comparator group. Causality for each individual event could not be definitively determined for these events, but factors supporting a possible association include the close temporal association with vaccination and lack of identification of alternate illness aetiologies. Although three patients had CHIK-LA co-administered with other vaccines, an association with the co-administered vaccines seems less likely because long-term safety experience with JE-VC and oral typhoid vaccine has not indicated any concern for cardiac or neurological events [9,10]. In addition, two patients had test results supportive of an association with vaccination with CHIK-LA, including one with chikungunya virus antibodies in cerebrospinal fluid and another with ribonucleic acid detected at an unusually long interval after vaccination [8,11].

Chikungunya virus can cause an acute illness with fever and severe arthralgia, with older adults at higher risk for severe illness and hospitalisation [12]. In addition, immunosenescence can affect an older person’s ability to adequately control replication of viruses, including live attenuated vaccine viruses as seen with yellow fever vaccine [13]. The observation of SAEs in older persons is unsurprising if SAEs after use of CHIK-LA are due to inadequate immune system control of replicating vaccine virus.

Limitations of this report include potentially incomplete and inconsistent available medical information, as can occur with any passive surveillance system. Despite our enhanced surveillance efforts, this might reflect healthcare providers not recommending tests, patients declining tests or unavailable or missing results when the medical records were provided. This limited a comprehensive assessment of some AEs. In addition, event rates are possibly misestimated because of inaccuracies in the numerator or denominator data. To ensure that adequate evaluations of SAEs following immunisation are possible, it is important that healthcare providers conduct thorough investigations, including chikungunya molecular or other testing as appropriate, and report events through AE reporting systems.

Based on findings from VAERS enhanced surveillance activities and discussions with the US Advisory Committee on Immunization Practices (ACIP) Chikungunya Vaccines Work Group, CDC alerted healthcare providers to the SAEs in early 2025. After review and discussion of the six SAEs at the April 2025 ACIP meeting, ACIP committee members voted to revise the traveller recommendations for CHIK-LA, including removing a previous recommendation specifically for use in older persons, and supported introducing age ≥ 65 years as a precaution for use of CHIK-LA (Box) [4]. A precaution indicates that in general, vaccination should be deferred but might be indicated if the benefits of protection from the vaccine outweigh the risks for an adverse event.

Box **US Advisory Committee on Immunization Practices revised recommendations for use of live attenuated chikungunya vaccine, April 2025**

The ACIP recommends the live attenuated chikungunya vaccine for persons aged ≥ 18 years travelling to a country or territory where there is a chikungunya outbreak.

In addition, the live attenuated chikungunya vaccine may be considered for persons aged ≥ 18 years travelling or taking up residence in a country or territory without an outbreak but with elevated risk for US travellers if planning travel for an extended period of time, e.g. 6 months or more.

ACIP: Advisory Committee on Immunization Practices; US: United States.The recommendation indicates vaccination is recommended for persons aged ≥18 years, but additional guidance from the Centers for Disease Control and Prevention indicates age ≥ 65 years is a precaution for use of the live attenuated vaccine. Similar recommendations were also approved by ACIP for use of a virus-like particle chikungunya vaccine (CHIK-VLP; manufactured by Bavarian Nordic as VIMKUNYA) in adolescents and adults aged ≥ 12 years; there is no restriction on CHIK-VLP use in older persons [18].

The following week, on 26 April, French health authorities noted that three SAEs, including one death, had occurred during the vaccination programme in Reunion Island that was undertaken to mitigate a large outbreak, and removed a recommendation for vaccination in persons aged ≥ 65 years [14]. On 7 May, the European Medicines Agency (EMA) reported a global total of 17 SAEs, including two deaths, in persons aged 62–89 years; this included the six SAEs in people living in the US and 11 in non-US persons. The EMA recommended a pause in CHIK-LA use among persons aged ≥ 65 years to allow investigation of the reports [15]. On 9 May, FDA and CDC recommended a pause in use in persons aged ≥ 60 years [16]. In Europe, the pause was lifted and updated guidance published in July notes that CHIK-LA is to be used only when there is a significant chikungunya risk and after careful consideration of the benefits and risks [17]. In the US, FDA lifted the pause on 6 August after completion of a benefit–risk assessment [5]. The FDA required updates to the CHIK-LA prescribing information, including text noting that the vaccine is only indicated for those at high risk of exposure to chikungunya virus, that vaccination is not advisable for most US travellers because of the low risk of chikungunya, that the decision to administer the vaccine should take into consideration an individual’s risk of severe or chronic disease outcomes if infected with chikungunya virus and risks of serious, severe or prolonged chikungunya-like illness caused by vaccination, and that individuals aged ≥ 65 years with one or more chronic medical conditions might have an increased risk for SAEs. 

## Conclusion

Monitoring vaccine safety after licensure is vital to detect any potential safety concerns not identified in pre-licensure clinical trials. Although causality for each of the six SAEs could not be definitively determined, detection of a general safety signal among older adults allowed timely public health action to be taken. Vaccination can prevent potentially severe chikungunya disease, so an individual assessment of risks and benefits for each traveller is important. Continued safety monitoring will ensure healthcare providers, public health officials, and the public have timely information if any future safety concern was to be identified. 

## Data Availability

Data from reports to the Vaccine Adverse Event Reporting System (VAERS) can be accessed at https://vaers.hhs.gov/data.html.
